# Health Care Professionals’ Experiences Regarding Facilitators of and Barriers to Sustained Use of Social Robot Ivy for People With Intellectual Disabilities: Qualitative Interview Study

**DOI:** 10.2196/74168

**Published:** 2025-09-10

**Authors:** Mark Steins, Claire Huijnen, Gaby Odekerken-Schröder, Dominik Mahr, Kars Mennens, Ramon Daniels, Frank Mathmann

**Affiliations:** 1 Department of Marketing and Supply Chain Management, Maastricht Center for Robots School of Business and Economics Maastricht University Maastricht The Netherlands; 2 School of Advertising, Marketing and Public Relations Faculty of Business and Law Queensland University of Technology Brisbane Australia; 3 Research Centre for Assistive Technology in Care Zuyd University of Applied Sciences Heerlen The Netherlands

**Keywords:** assistive technology, social robots, health care innovation, implementation, adaptive implementation, intellectual disability care, barriers, facilitators, field study, technology continuance, adoption

## Abstract

**Background:**

Labor shortages in health care pose significant challenges to sustaining high-quality care for people with intellectual disabilities. Social robots show promise in supporting both people with intellectual disabilities and their health care professionals; yet, few are fully developed and embedded in productive care environments. Implementation of such technologies is inherently complex, requiring careful examination of facilitators and barriers influencing sustained use.

**Objective:**

This research aimed to evaluate the value creation and implementation of social robots for people with intellectual disabilities and health care professionals, examining facilitators of and barriers to sustained use across 6 care organizations.

**Methods:**

A qualitative field study was conducted involving 19 cases of robot implementation across 6 care organizations in the Netherlands; each case consisted of people with intellectual disabilities (clients) and the involved health care professionals. The study examined actual robot deployment in daily care practice between April 2023 and October 2023. Semistructured interviews were conducted with health care professionals after 2 months of implementation. Analysis followed a thematic approach guided by the nonadoption, abandonment, scale-up, spread, and sustainability framework and a model for tracing facilitators of and barriers to the adaptive implementation of the robot. Facilitators were classified as key drivers (complex), enablers (complicated), and minor benefits (simple), whereas barriers were categorized as deal-breakers (complex), obstacles (complicated), and minor hurdles (simple). The robots’ sustained use (ie, robot use continuance at 2 months after implementation) served as a key indicator of success.

**Results:**

After 2 months, robot use continued in 63% (12/19) of cases. For successful cases, the robot created distinct value among both clients (enhanced daily structure, improved emotional well-being through nonjudgmental interactions, and increased independence) and health care professionals (reduced workload through automation, improved quality of client interactions, and reduced emotional burden). Client characteristics (cognitive capabilities and care predictability), health care professional factors (available time and digital competency), contextual conditions (timing and connectivity), and organizational support (training and resources) influenced sustained use. Main implementation barriers included complex or unpredictable care needs, insufficient programming time, and contextual factors affecting care environments.

**Conclusions:**

The findings inform long-term care organizations on the implementation and value of the sustained use of social robots for both people with intellectual disabilities and their caregivers. Social robot Ivy demonstrates potential for supporting care delivery to people with intellectual disabilities when implemented under appropriate conditions. Success requires careful matching of robot capabilities with client needs, sufficient time and support for health care professionals, and stable care environments. Future research should examine longer-term sustainability and integrate direct client feedback.

## Introduction

### Background

Labor shortages in the health care sector pose significant challenges to sustaining high-quality care worldwide. According to the World Health Organization, a global shortage of 10 million health care workers is projected by 2030, with the quality of care heavily dependent on their availability, accessibility, and expertise [1]. In mental health care, the growing demand for care services for people with intellectual disabilities further amplifies the challenges and places additional strain on already overstretched resources [2]. This pressing reality threatens the sustainability of high-quality, person-centered care for people with intellectual disabilities. In response, there is growing interest in technological solutions that could help address staffing shortages while maintaining the envisioned care.

Reflecting this growing interest in technological solutions, intelligent assistive technologies (IATs) are increasingly being used to support care for people with intellectual disabilities [3]. These empowering technologies aimed at maintaining or improving an individual’s functioning and independence and, thereby, promoting their well-being include a range of digital devices, from smart home systems and tablets to wearable devices and humanoid robots [4,5]. Among these technologies, social robots have emerged as a particularly promising innovation. These robots have the potential to offer support to various target groups with complex needs, addressing a wide range of domains in daily life, including communication, social interaction, interpersonal relationships, emotional well-being, and functioning in everyday contexts [6,7].

Systematic reviews in mental health care suggest that social robots have the potential to improve mood, social interaction, and activity participation for individuals with intellectual disabilities through both verbal and nonverbal interactions [8,9]. However, challenges such as unmet expectations, time constraints, limited technical capabilities, and ethical concerns have been reported, highlighting the need to carefully manage implementation processes to ensure long-term success [10]. These preliminary insights underscore a pressing need for empirical studies that examine both implementation processes and outcomes to better understand the potential role of social robots in mental health care, address implementation enablers and barriers, and determine their value for people with intellectual disabilities and their personal caregivers. So far, few robots are fully designed and deployed in long-term care, and hence, the opportunities to conduct this necessary research have been limited.

One notable example of a social robot that is already operational in the care process is Ivy, recently implemented as part of a regional collaborative initiative across 6 care organizations in the Netherlands [11,12]. Ivy is designed to support and accompany people with intellectual disabilities in their daily lives, whether in care facilities or at home, by stimulating cognitive functions through personalized interactions. By doing so, Ivy also intends to support health care professionals by enhancing efficiency, enriching client care, and contributing to greater work satisfaction. Health care professionals can customize Ivy to tailor its support to each client’s needs, providing assistance with their daily structure and personalized reminders for appointments and medication and facilitating social interactions. The robot communicates through time-based preprogrammed verbal interactions and displays supportive visual information on its screen.

The care organizations decided to introduce these social robots to their clients with intellectual disabilities due to Ivy’s intended impact on both clients and health care professionals. Its real-world deployment offers a unique opportunity to examine the value created by the robot for people with intellectual disabilities and their professional caregivers, as well as the facilitators and barriers influencing its ability to create value for these 2 key stakeholders.

Implementing robots in health care and long-term care settings is inherently complex [11], with little evidence guiding their use [13]. To understand the implementation process and outcomes, we drew upon 2 complementary theoretical frameworks. First, the nonadoption, abandonment, scale-up, spread, and sustainability (NASSS) framework helps analyze implementation challenges across multiple interacting domains: the condition, the technology, the value proposition, the adopter system (comprising professional staff and clients), the organization, the wider context, and the interaction among these domains over time [14]. This framework categorizes implementation challenges as simple (straightforward, predictable barriers that are easily addressed), complicated (multiple interacting components requiring significant work to overcome), or complex (dynamic, unpredictable barriers that may lead to abandonment if not effectively managed).

Second, we used the theoretical model by Meiland et al [15] for tracing facilitators and barriers in the adaptive implementation of innovations in care settings. This model examines implementation across 4 phases (preconditions, preparation, execution, and continuation) and 3 levels (micro, meso, and macro). While preconditions and preparation were addressed before our study, we focused specifically on the execution and continuation phases at the micro level, examining how the end users, clients with intellectual disabilities and professional caregivers, experienced and used robot Ivy over the first 2 months of implementation.

### Objectives

Together, these frameworks guided our investigation of both facilitators of and barriers to successful adaptive implementation. To begin, the NASSS framework allowed us to classify barriers by their level of complexity and likely impact on sustained use. By applying the same classification framework to facilitators as we did to barriers, we could better assess their relative impact on the success of implementation. We analyzed these facilitators and barriers separately for each key stakeholder (ie, people with intellectual disabilities and health care professionals) while also considering contextual factors unique to each implementation case and organizational factors. In the case of social robot Ivy, this categorization can help identify which facilitators to strengthen or further leverage, as well as which barriers to continued robot use are straightforward and manageable versus those that require more sophisticated support strategies. In this study, we applied these complementary frameworks to analyze the adaptive implementation of the social robot Ivy in care for people with intellectual disabilities, focusing in particular on microlevel factors within the adopter system domain (people with intellectual disabilities and health care professionals) and the technology and value proposition domain (perceived value for these users). However, as we investigated both the execution and continuation phases of implementation of social robots across 19 people with intellectual disabilities and their professional caregivers in actual productive care environments, our analysis also included contextual and organizational factors from the caregiver’s perspective, offering implications for the broader health care organization. Through semistructured in-depth interviews with health care professionals (in this study also referred to as *professional caregivers*) after 2 months of implementation, we generated an understanding of facilitators of and barriers to successful implementation, categorizing them by their level of impact on sustained use. We investigated the staff perspective as their perceptions play a key role in the adoption of social robots [16,17]. Our research questions were as follows:

1. What value does sustained use of social robot Ivy create for people with intellectual disabilities and health care professionals?
2. What facilitators and barriers influence the continuation versus abandonment of use of social robot Ivy during the execution and continuation phases of implementation? What client and health care professional characteristics influence sustained use? What contextual and organizational factors affect implementation success?

## Methods

### Study Design, Participants, and Setting

This study was conducted between April 2023 and October 2023 across 6 care organizations in the southeast region of the Netherlands. A qualitative design was used to assess the added value and user-friendliness of the social robot Ivy, as well as to identify facilitators and barriers to its sustained use for people with intellectual disabilities receiving care under the Long-term Care Act (Dutch: *Wet langdurige zorg*) [18]. This study followed 19 robot Ivy devices implemented within these care settings. Data were collected through semistructured in-depth interviews with key stakeholders. Two distinct selection processes took place. First, care organizations independently selected clients for robot implementation based on clinical eligibility criteria. Second, we purposefully sampled those health care professionals who were directly responsible for introducing and integrating robot Ivy into client care for interviews. While multiple stakeholder groups were involved in the implementation of the robot—including clients, health care professionals, and relatives or legal representatives—this study specifically focused on the perspectives of health care professionals. As they played a central role in introducing and integrating Ivy into daily care, their firsthand insights provided the most consistent and detailed account of the implementation process. Clients’ cognitive limitations posed challenges for conducting meaningful or extensive in-depth interviews, and relatives were generally less engaged in the robot’s daily use. Therefore, we recruited health care professionals who were directly responsible for introducing and integrating robot Ivy into client care. Participants were selected through purposive sampling, ensuring a diverse representation of perspectives across the participating organizations. The qualitative approach allowed for an in-depth exploration of how Ivy was integrated into daily care routines, the perceived value and challenges, and the contextual factors influencing long-term adoption. Interviews with health care professionals took place 2 months after the robot introduction to the client.

The clients had moderate to severe intellectual disabilities requiring 24-hour care or continuous supervision. This population includes individuals with a congenital or later-developed disorder in intellectual functioning who also experience limitations in adaptive behavior, such as conceptual, social, and practical skills, as described by Schalock et al [19]. Throughout this paper, we use the terms *people with intellectual disabilities* and *clients* interchangeably to refer to people with intellectual disabilities, with *client* denoting their status as recipients of care services. For context, clients were eligible to use the robot if they (1) had sufficient visual, auditory, and cognitive capabilities to process information from the robot; (2) could maintain appropriate attention to interact with the robot; and (3) were residents at one of the participating care organizations. Exclusion criteria included blindness, deafness, or severe sensory processing disorders that would prevent meaningful interaction with the robot.

In total, the 19 unique cases studied represent the total population for robot implementation that took place during the data collection period, each case involving a client and their health care professionals. For each case, we conducted interviews with the health care professionals 2 months after the robot was introduced. The participating organizations in the Social Robotics program determined this 2-month implementation period as sufficient to move beyond novelty effects and assess whether the robot had become integrated into the client’s daily life and routine care practices. With robot Ivy typically providing multiple daily interactions (eg, morning routines, medication reminders, and activity prompts), the 2-month period encompassed approximately 300 to 900 discrete interaction moments per client, providing substantial evidence of implementation patterns while minimizing the potential influence of external organizational changes unrelated to the study.

### The Technology: Robot Ivy

**Figure 1:**
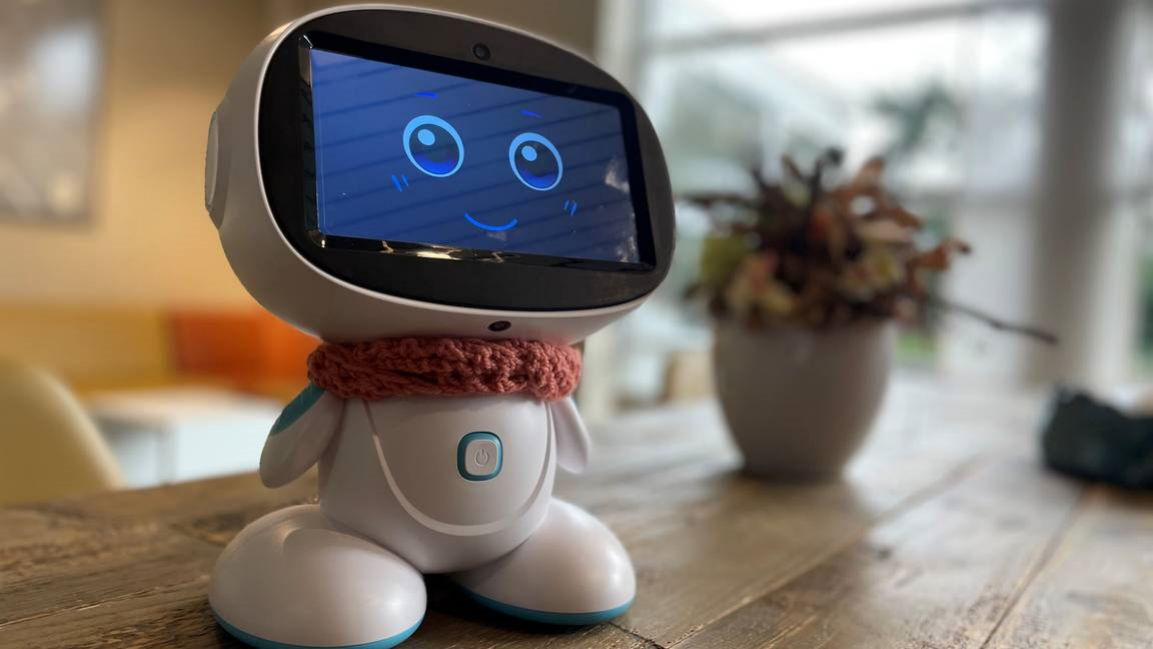
Robot Ivy.

Robot Ivy (Figure 1) is a lightweight, small-size stationary social robot that health care professionals can customize through a browser-based platform to support each client’s needs, providing clients assistance with their daily structure and personalized reminders for appointments and medication and facilitating social interactions [12,20]. As such, the technology consists of 2 distinct interfaces, namely, the physical robot that faces people with intellectual disabilities and health care professionals and the browser-based platform where health care professionals set up and customize client interactions.

The client-facing interface consists of the physical robot itself, which communicates with clients through a humanlike voice that can be accompanied by pictograms and text on the robot’s touch screen. While Ivy communicates through spoken messages, it does not respond to voice commands. Clients can interact with Ivy through the touch screen and receive various types of personalized support, such as daily structure, appointment and medication reminders, cognitive stimulation, entertainment, social interaction, and companionship. Whenever the robot is not offering an interaction to the client, it displays a friendly, dynamic face to maintain an engaging and approachable presence (Figure 1). The robot requires its own access point for internet connectivity and operates independently from existing health care information systems. Ivy executes preprogrammed tasks without independent decision-making capabilities.

Health care professionals design and customize client interactions through the separate browser-based platform, which allows them to tailor interactions specifically to the needs of individual clients. Interactions can either be scheduled at specific time slots for plannable care (eg, a morning routine) or made available for proactive activation by clients (eg, when a client wants the robot to tell a joke or help communicate on their behalf). This interface allows health care professionals to configure and continuously adapt various types of interactions, including reminders, questions, stories, display of photos and music playback using voice-only communications, voice with touch screen response options, or voice with pictogram responses.

### Data Collection

We investigated 19 cases of robot implementation, meaning 19 individual people with intellectual disabilities who made use of social robot Ivy. Data were collected through semistructured in-depth interviews with the health care professionals responsible in those cases, which were conducted 2 months after robot Ivy was introduced to their respective client. The interviews explored professionals’ experiences with implementing the robot, perceived value and user-friendliness for both staff and clients, and factors influencing whether the robot’s use was continued or discontinued after the initial implementation period.

Two researchers (authors MS and CH) conducted the interviews, lasting approximately 45 to 60 minutes each. Most interviews were conducted face-to-face and audio recorded. Some interviews were conducted via Microsoft Teams videoconferencing due to scheduling constraints and were video recorded. While most interviews were one-on-one, 4 cases involved multiple professionals in group interviews (where multiple professionals were involved in implementing Ivy for that client). The 2-month timing allowed for a systematic and meaningful evaluation of the experiences of health care professionals and the identification of key facilitators and barriers influencing sustained use versus nonadoption or abandonment, in line with our theoretical frameworks (the NASSS framework and the model by Meiland et al [15]).

### Data Analysis

All interviews were audio or video recorded and transcribed verbatim. A structured thematic analysis was conducted guided by the implementation model by Meiland et al [15] and the NASSS framework [14]. Codes for facilitators and barriers per stakeholder (eg, people with intellectual disabilities or clients and health care professionals), as well as contextual and organizational factors, were created. The types of barriers were derived from the NASSS framework [14]—deal-breakers (high impact or complex), obstacles (medium impact or complicated), and minor hurdles (low impact or simple). Facilitators were constructed to mirror the severity of impact that these barriers have on sustained use (high, medium, or low) and were categorized as key drivers, enablers, and minor boosters.

To ensure coding reliability and develop a shared understanding of the data, 3 researchers independently coded 5 interview transcripts. Through detailed discussion of discrepancies, the team developed a consistent interpretation of the coding framework, which guided 1 researcher in coding the remaining transcripts.

Data saturation was achieved within our available sample when no new facilitators and barriers emerged from subsequent interviews, with recurring themes appearing across cases regardless of client diagnosis complexity or organizational setting. While our sample was determined by the robot implementations available during the data collection period, including both sustained use (12/19, 63%) and discontinued (7/19, 37%) cases ensured comprehensive coverage of the range of implementation experiences observed until thematic saturation was reached within this dataset.

### Ethical Considerations

This study was approved by the Medical Ethics Review Committee (Dutch: *Medisch Ethische Toetsingscommissie Zuyderland-Zuyd*; Zuyderland Medical Center) [21] and is known under approval METCZ20230028. Written informed consent was obtained from all participants. Participants did not receive monetary compensation for taking part in this study. This decision was made to minimize potential coercion, particularly given the vulnerable nature of the client population and the existing care relationships between participants and their health care organizations. The voluntary nature of participation was emphasized throughout the recruitment and consent process, and participants were informed that they could withdraw from the study at any time without consequences for their employment or care. Data were deidentified during the coding and analysis process to protect participant privacy. Audio recordings were transcribed with all identifying information removed, and participants were assigned numerical codes. The data storage infrastructure complies with institutional security standards, including encrypted storage, regular backup procedures, and access restricted to authorized research team members.

## Results

### Participants

In total, 19 interviews were conducted with 23 health care professionals, all of whom were directly involved in implementing 19 social robot Ivy devices as part of their clients’ care. Of these 23 professionals, 19 (83%) identified as female, and 4 (17%) identified as male. Table 1 presents the distribution of research participants across cases nested within the 6 care organizations. The presentation of cases in this paper follows a chronological order, representing the sequence of data collection. One professional (participant 1) took part in 2 separate cases as this caregiver was responsible for the direct care of 2 clients using the robot. While most interviews were conducted one-on-one, in 4 cases (cases 1, 6, 9, and 12), multiple professionals participated in group interviews as they were all involved in the implementation of social robot Ivy for a client in those cases.

**Table 1:** Research participants across cases and care organizations.

Case	Care organization	Research participants
1	A	1 and 2
2	F	3
3	F	4
4	B	5
5	C	6
6	D	7 and 8
7	C	9
8	A	1
9	C	10 and 11
10	A	12
11	F	13
12	F	14, 15, and 16
13	F	17
14	B	18
15	E	19
16	E	20
17	A	21
18	A	22
19	D	23

### Sustained Use of Social Robot Ivy

Answering the first research question, this section presents our findings on the value of the sustained use of social robot Ivy based on health care professionals’ perspectives after 2 months of implementation. The robot demonstrated distinct value for both people with intellectual disabilities and health care professionals.

#### Value for People With Intellectual Disabilities

For people with intellectual disabilities, the primary value emerged in three areas: (1) enhanced daily structure through consistent reminders and routines, (2) improved emotional well-being through nonjudgmental interactions and companionship, and (3) increased independence in daily activities through empowerment.

#### Enhanced Daily Structure

As professionals noted, the robot supported in providing an enhanced daily structure, as illustrated by several quotes:

Using Ivy has enabled the client to independently start their day, which increases their self-confidence and saves us as professionals time and energy.

Participant 1

The robot provides structure which gives her more independence, and her emotional well-being has improved. The robot has become like a friend to her.

Participant 3

#### Improved Emotional Well-Being

The robot’s value for emotional well-being was particularly evident through its role as a companion and source of nonjudgmental support. The robot’s emotion-free and nonjudgmental communication proved particularly valuable for clients who tend to interpret human interactions negatively or seek excessive validation. One professional explained the following:

Ivy is just the same every day, she doesn’t need to shop around between me or a colleague if she gets a “no” from one person and wants a “yes” from another. So, I think that provides a lot of peace. And Ivy is just the same every day, she can’t negotiate with it. I think that also provides a piece of clarity and peace.

Participant 8

This consistent, neutral communication style helped reduce anxiety-inducing dynamics that could arise from varying responses from different care professionals while providing clear structure and boundaries. Health care professionals observed that the robot contributed to building clients’ self-confidence, reducing restlessness, and promoting emotional attachment.

#### Increased Independence Through Empowerment

One health care professional stated the following:

We do this because he is very proud to mention that care staff no longer needs to come in with him, he is very proud that he can now do this independently. So, his self-confidence is growing.

Participant 1

The robot provided support in daily activities such as getting up on time, brushing teeth, showering, eating, drinking, and taking medication, which increased clients’ control over their daily routines and enabled them to perform certain tasks independently. Moreover, beyond fostering greater independence for people with intellectual disabilities, the increased autonomy supported by the robot also had meaningful implications for both clients and caregivers. One health care professional highlighted how the robot reduced the need for constant reminders to the client, reinforcing the client’s sense of control and dignity:

Of course, we are not always with her, and the fact that we no longer have to give certain reminders helps. I’m not going to remind her to take her medicine, if I know she already got the reminder from Ivy. That would be redundant and unnecessary...It gives her control and autonomy, and with that, a sense of self-worth.

Participant 3

Another health care professional described how the robot actively encouraged social participation:

We notice that the robot encourages him to join the group, to ask others what his needs are.

Participant 7

#### Value for Health Care Professionals

For health care professionals, the value manifested in several ways: (1) reduced workload through automation of routine tasks, (2) improved quality of client interactions by freeing up time for meaningful engagement, (3) reduced emotional burden by having the robot absorb repetitive client interactions, and (4) enhanced work satisfaction through more efficient care delivery.

#### Reduced Workload Through Automation of Routine Tasks

Health care professionals reported that Ivy helped them achieve a better balance between delivering efficient care and maintaining meaningful human interactions. One professional explained the following:

For us as professionals, it’s about the tasks it performs without us having to repeat instructions to the client over and over. This allows us to be with the client at other moments, and instead of saying “do this, do that,” we can say “come, let’s have a coffee together.” Ivy has already handled the rest—that’s our gain.

Participant 13

#### Improved Quality of Client Interactions by Freeing Up Time for Meaningful Engagement

Furthermore, professionals highlighted how the robot reduced workload while improving client relationships:

Yes, the moments that Ivy takes over, like waking up and medication reminders, reduce our workload. Ivy also provides structure and clarity, which makes the relationship with the client more stable.

Participant 7

#### Reduced Emotional Burden

The robot’s ability to handle repetitive client interactions without emotional fatigue was particularly valuable. One professional noted the following:

Where staff might have an emotional reaction after hearing the same story multiple times...that will never happen with Ivy.

Participant 9

#### Enhanced Work Satisfaction Through More Efficient Care Delivery

In addition, the robot’s 24/7 availability provided valuable support during staff absences, ensuring continuity of care and allowing professionals to focus more on personalized support. In the quote by research participant 3 in the Increased Independence Through Empowerment section, this participant highlighted how the robot reduced the caregivers’ need to give constant reminders, reinforcing the client’s sense of control and dignity.

In some cases, the robot contributed to a more stable environment and improved client well-being. As a result, staff spent less time managing crises, enabling them to prioritize meaningful engagement and individualized care for clients. For health care professionals, benefits emerged when the robot successfully engaged clients. During these moments of engagement, staff gained opportunities to attend to other care tasks. One professional stated the following:

In those ten-fifteen minutes you are again a bit more available for others. So, I do see the advantages in that.

Participant 15

However, the extent of value creation and perceived value for clients as well as health care professionals varied significantly across cases. Health care professionals defined client-specific care goals for the robot before implementation, with these goals fully achieved in 26% (5/19) of cases, partially achieved in 32% (6/19) of cases, and not achieved in 42% (8/19) of cases. After 2 months, the robot remained in use for 63% (12/19) of the clients, whereas it was discontinued for 37% (7/19) of the clients, demonstrating that certain conditions need to be met for sustained use. Having multiple cases of both outcomes proved highly valuable for investigating facilitators and barriers, enabling a more nuanced understanding of the factors influencing the sustained use of the robot.

### Facilitators of and Barriers to Implementation of Social Robot Ivy

#### Overview

Following our theoretical frameworks, we categorized both facilitating and impeding factors affecting the sustained use of robot Ivy. Regarding barriers, following the NASSS framework, we classified them into 3 categories: deal-breakers, which often lead to nonadoption or abandonment; obstacles, which require significant effort or time to overcome; and minor hurdles, which are relatively easy to address. To mirror the impact of these barrier levels, facilitators were categorized as key drivers, enablers, and minor boosters.

In the following sections, guided by the second set of our research questions, we present our findings across four key areas that emerged from the data: (1) client-related factors, examining characteristics and needs of people with intellectual disabilities that influence robot use; (2) health care professional–related factors, focusing on staff experiences and capabilities; (3) contextual factors, exploring implementation conditions across the 19 cases; and (4) organizational support factors, addressing meso-level elements that enable or hinder implementation of social robot Ivy in the micro environment with end users. Textboxes 1-4 provide a comprehensive overview of these client-, health care professional–, context-, and organizational-related facilitators and barriers. The following sections present findings on these 4 key areas, highlighting frequently occurring and impactful facilitators and barriers supported by illustrative quotes from our interviews with health care professionals. While our research primarily focused on microlevel implementation factors related to the end users (clients and their health care professionals), the interviews with health care professionals also revealed broader organizational, meso-level influences on sustained use of social robot Ivy and, thus, implementation success.

**Client-related facilitators and barriers influencing the sustained use of robot Ivy.**

**Facilitators**

- Key drivers: sufficient cognitive and sensory capabilities to process robot interactions (facilitator 1.1); ability to maintain attention during robot interactions (facilitator 1.2); need for structure and predictable routines (facilitator 1.3); and receptiveness to neutral, consistent communication (facilitator 1.4)
- Enablers: positive initial response to the robot (facilitator 1.5); interest in and openness to the robot (facilitator 1.6); robot as a buddy, trust, and confiding in and sharing with the robot (facilitator 1.7); and client involvement in programming the robot (facilitator 1.8)
- Minor boosters: previous experience with technology (facilitator 1.9) and ability to form emotional connections with the robot (facilitator 1.10)

**Barriers**

- Deal-breakers: complex, unpredictable care needs requiring frequent adjustments (barrier 1.1), inability to process or follow robot instructions independently (barrier 1.2), strong resistance to technology adoption (barrier 1.3), severe cognitive limitations affecting comprehension (barrier 1.4), a mismatch between a client’s expectations and robot functionality (barrier 1.5), and fluctuating client well-being and health conditions affecting engagement (barrier 1.6)
- Obstacles: limited mobility restricting robot access (barrier 1.7), difficulty maintaining interest over time (barrier 1.8), and need for constant staff prompting to engage with the robot (barrier 1.9)
- Minor hurdles: initial adjustment period to the robot (barrier 1.10), technical difficulties with touch screen operation and interaction pace (barrier 1.11), and occasional misunderstanding of robot instructions (barrier 1.12)

**Health care professional–related facilitators and barriers influencing the sustained use of robot Ivy.**

**Facilitators**

- Key drivers: willingness to learn and adapt to new technology (facilitator 2.1), ability to program basic client interactions (facilitator 2.2), additional dedicated time for robot setup and adaptation (facilitator 2.3), and understanding of individual client needs (facilitator 2.4)
- Enablers: team-wide commitment to robot implementation (facilitator 2.5), regular evaluation and adjustment of robot interactions (facilitator 2.6), creativity and ability to personalize robot interactions based on client knowledge (facilitator 2.7), and adding a mobile app for the platform for more agile programming (in addition to the browser-based version; facilitator 2.8)
- Minor boosters: previous experience with (health care) technology (facilitator 2.9) and enthusiasm for innovative care solutions (facilitator 2.10)

**Barriers**

- Deal-breakers: insufficient time for robot setup and adaptation (barrier 2.1) and high staff turnover (barrier 2.2)
- Obstacles: time constraints during busy shifts (barrier 2.3); inconsistent use across team members, often caused by a lack of knowledge and skill or a perceived lack of effectiveness of the robot (barrier 2.4); challenges in programming complex interactions (barrier 2.5); and use of robot intervention in care not part of DNA yet (barrier 2.6)
- Minor hurdles: initial learning curve with programming interface (barrier 2.7) and need for regular updates and adjustments (barrier 2.8)

**Contextual facilitators and barriers influencing the sustained use of robot Ivy.**

**Facilitators**

- Key drivers: clear integration with existing care routines (integral part; facilitator 3.1)
- Enablers: support from family and caregivers (facilitator 3.2)

**Barriers**

- Deal-breakers: structural issues with connectivity and robot technology (barrier 3.1) and poor timing of implementation (barrier 3.2)
- Obstacles: coordination challenges during holiday periods and limited capacity of staff across shifts (barrier 3.3)
- Minor hurdles: occasional technical glitches (barrier 3.4)

**Organizational facilitators and barriers influencing the sustained use of robot Ivy.**

**Facilitators**

- Key drivers: management support for implementation (time and training; facilitator 4.1)
- Enablers: dedicated implementation team (facilitator 4.2), regular training opportunities (facilitator 4.3), and shared learning opportunities across teams (documentation of successful use cases; facilitator 4.4)
- Minor boosters: recognition of staff efforts in robot implementation (facilitator 4.5)

**Barriers**

- Deal-breakers: lack of organizational commitment (barrier 4.1) and diverging from implementation road map (introducing a robot to the client before staff training in the use of the robot; barrier 4.2)
- Obstacles: unclear implementation responsibilities and islands (isolated initiatives) fragmented within the organization (barrier 4.3)

#### Client-Related Factors Influencing Sustained Use of Robot Ivy

Client characteristics and behaviors emerged as crucial determinants of successful robot implementation. All interviews on the 19 cases revealed that certain client traits consistently facilitated or impeded sustained robot use, particularly regarding cognitive capabilities, care need predictability, and receptiveness to technological intervention. These characteristics significantly influenced whether the robot could effectively support clients’ daily activities and emotional well-being. The following paragraphs elaborate on frequently mentioned and impactful factors across the interviews, with numbers corresponding to their listing in Textbox 1.

As highlighted by several health care professionals, matching client needs with robot capabilities was essential for successful implementation. Most professionals emphasized key driver 1.1—sufficient cognitive capabilities:

There are moments that Ivy gives an instruction and then the client executes that, but there are also still many moments that it doesn’t work. We are still looking at what exactly this is due to? Does the client not hear it or does the transition still pose difficulties?

Participant 22

Impactful deal-breakers regarding the sustained use of the robot included clients experiencing distress, mental health challenges, or instability (barrier 1.6), resulting in reduced or no capacity to engage with Ivy. This was observed in clients at risk of epileptic seizures, relapsing into substance use, displaying compulsive behaviors, or experiencing negative emotions. One professional noted the following:

Client XYZ is currently doing so poorly that he’s not open to anything new. But once that peak of tension subsides, he becomes more accessible, and we can work with him. That’s when Ivy has the greatest chance of succeeding.

Participant 5

Another stated the following:

We are now dealing with interventions, bed, bath, food and after that we might be able to work with Ivy again if the entry point comes.

Participant 6

Another dominant theme throughout the interviews was the importance of setting accurate expectations regarding the robot’s capabilities. Multiple interviews mentioned enabler 1.5—positive initial response to the robot—as most clients were comfortable with Ivy’s one-way communication style given that their primary need was expression rather than reciprocal conversation. Ivy’s role as a passive listener proved sufficient for clients to experience emotional unburdening even without receiving a response. One care professional described the following:

He doesn’t need an answer, but just the part where he feels like, okay, I’ve let it out.

Participant 11

However, health care professionals also indicated that, for some clients, Ivy’s interactivity seemed too restricted, in some cases leading to discontinued use. This represents barrier 1.5—a mismatch between client expectations and robot functionality—which turned out to be a deal-breaker for sustained use. The robot’s inability to engage in 2-way conversations limited its effectiveness in addressing feelings of loneliness. One care professional noted the following:

...she lost interest as she had expected Ivy to be more socially interactive.

Participant 17

Moreover, professionals emphasized the challenges of complex and unpredictable care needs (deal-breaker 1.1):

In reality, care for clients is not so plannable as initially assessed. This makes timing of interactions extremely difficult. Especially when interaction follow up fast, when one interaction’s timing goes wrong, the whole day gets out of sync.

Participant 5

In contrast, facilitator 1.8, early client engagement in robot programming, emerged as an enabler for sustained use. Health care professionals emphasized that involving clients in customizing Ivy’s functionalities enhanced their sense of ownership of the robot. One professional noted the following:

We involve the clients in everything. We don’t do anything without them—they lead the process.

Participant 3

This collaborative approach helped ensure that the robot’s capabilities aligned with the specific needs and preferences of people with intellectual disabilities. However, it is important to note that, across the 19 cases, not all people with intellectual disabilities were capable of such engagement due to cognitive limitations, highlighting the need for tailored implementation approaches.

#### Health Care Professional–Related Factors Influencing Sustained Use of Robot Ivy

Health care professionals’ attitudes, digital competencies, and available time emerged as critical factors influencing successful robot implementation. Their ability to effectively set up and maintain the robot, combined with their willingness to integrate it into daily care routines and the robot’s embeddedness in the care delivery, significantly impacted implementation success. The following paragraphs present some striking facilitators and barriers from Textbox 2, as evidenced by their frequent occurrence in successful versus discontinued cases.

The most critical barrier affecting sustained use was deal-breaker 2.1—insufficient time for programming and adapting to the robot over time. One professional explained the following:

At the beginning, I actually experienced more work instead of less, because it was mainly figuring out how to set those interactions.

Participant 13

Another interviewee noted the ongoing time investment required:

I have to plan time to be able to do that...Nothing can be done in between...It takes time to really program it well.

Participant 17

Key drivers 2.1 and 2.2—willingness to learn and adapt to new technology and the ability to program basic client interactions—emerged as essential facilitators in cases of successful implementation, as one professional noted:

I find it really exciting to explore new things and see what’s possible...I enjoy figuring out creative ways to make it work for a specific case. At first, programming was a bit tricky because I didn’t have the right permissions in the system, but once that was sorted out, it became much easier. Over time, I learned the steps, and now I know exactly what to do—it just takes practice. The more you use it, the faster and easier it gets.

Participant 17

However, obstacle 2.4—inconsistent use across team members—often undermined these positive factors, stemming from varying levels of knowledge and skill or perceived lack of effectiveness of the robot. One health care professional noted the following:

If the client does not respond to the robot or any of the robot interactions...so, there is no match or success...people [referring to colleagues] also become less motivated to use the robot and program interactions...When it is visibly successful and truly benefits the client, then the motivation is there for the staff as well, because it has a certain impact.

Participant 1

#### Contextual Factors Influencing Sustained Use of Robot Ivy

Implementation environments varied significantly across the 19 cases, encompassing differences in care settings, team dynamics, and implementation timing. These contextual elements proved crucial in determining implementation success, particularly regarding the stability of the care environment and availability of support resources. Textbox 3 summarizes the key contextual facilitators and barriers identified across cases. In the following paragraphs, we elaborate on these factors.

Health care professionals highlighted how deal-breaker 3.2—the timing of implementation—was affected by contextual factors related to organizational challenges such as sick leave. These temporary organizational challenges were also highlighted:

At the moment, it’s extremely restless in the living room. We have a lot of sick clients and colleagues, a lot of people who have decided to leave. So, there’s just a lot of unrest. A lot of unfamiliar staff that we’ve had to fly in, freelancers [in Dutch: ZZP’ers] because we just can’t handle it ourselves anymore...And I really notice that permanent staff, such as the colleagues doing the night shift and other fixed team members, use the robot and program it for the client. But now, due to the unrest in the team, working with lots of freelancers, it’s starting to be forgotten again.

Participant 20

In line with this, another professional reflected on other circumstances that caused coordination challenges (obstacle 3.3):

When Ivy arrived, a colleague of mine set it up. She also programmed the reminders for the client at that time. So, I did reach out to her and asked, “Hey, can you help me with this?” At one point, we tried to do it over the phone because meeting in person wasn’t possible. But the reality is, over the past few months, we’ve been dealing with extra workload due to a lot of staff being sick. We have a few colleagues on long-term sick leave, and there have also been vacations. So, in that sense, scheduling was also quite challenging.

Participant 4

#### Organizational Support Factors Influencing Sustained Use of Robot Ivy

While this research primarily focused on microlevel implementation factors, health care professionals’ experiences revealed that organizational support was crucial for successful implementation. Although we did not directly investigate organizational implementation strategies, professionals’ reflections highlighted how organizational factors enabled or hindered their ability to effectively use the robot in client care. The following paragraphs present further context for some of the frequently occurring organizational factors from Textbox 4.

Our analysis revealed a hierarchy of organizational support elements. Key driver 4.1—management support through training provision and resource allocation—proved essential. Training effectiveness varied significantly across organizations. When properly implemented, training sessions combined with accessible support channels enhanced professionals’ confidence and capability. One professional noted the following:

The training, the help desk. We can go anywhere with our questions. The facilities. We have a space to literally practice. And the people—I think they are easy to reach.

Participant 6

These essential elements facilitated sustained use of the robot. However, inconsistent training deployment created significant barriers. This was evident in one professional’s observation:

We should have immediately trained the whole team at once in how the system works instead of me having to do it staff member by staff member.

Participant 1

Resource allocation constituted another crucial organizational factor. While some organizations provided dedicated implementation time (enabler 4.2), others required integration into existing workflows. One professional described the following:

I close the office door and take a moment to focus, away from the residents. Staying an extra hour after my shift just to work on the robot would actually make things much easier. Right now, I have to manage it during my shift, and just when I spend 10 minutes on it, another resident needs my attention.

Participant 21

Cases that succeeded in implementation typically exhibited strong organizational commitment characterized by dedicated implementation teams, regular training opportunities, and thorough documentation of successful use cases (enablers 4.1-4.3). However, findings show that deal-breakers such as a lack of organizational commitment (4.1) and failure to adhere to the implementation road map (4.2) jeopardized the sustained use of the technology. In one case in which use of the robot was discontinued, a care professional shared the following:

I wasn’t there when Ivy was first introduced to us colleagues...so, I assume that a training on how the robot works could have helped. I also heard from colleagues that more people had the same issue. If you have to figure everything out on your own, it becomes very time-consuming, especially in a hectic period like this.

Participant 4

## Discussion

### Principal Findings

The sustained use of social robot Ivy in 63% (12/19) of cases demonstrates both the potential and challenges of integrating social robots into disability care. Building on previous research on IATs in disability care [3,5], this study examined the value of sustained use of social robot Ivy for people with intellectual disabilities and their health care professionals across 19 cases. The mixed results—where robot use continued in 63% (12/19) of cases but stopped in 37% (7/19) of cases—highlight the complex process of adopting technology in long-term care for people with intellectual disabilities. Our analysis revealed 3 key findings. First, in cases in which implementation was successful, social robot Ivy created distinct value for both clients and health care professionals through three key mechanisms: (1) the robot’s consistent and nonjudgmental nature provided structure while reducing emotional burden—supporting clients’ independence and well-being while allowing professionals to focus on meaningful interactions rather than repetitive tasks; (2) the automation of routine activities enhanced efficiency, enabling more person-centered care delivery; and (3) successful implementation appeared to create a virtuous cycle in which increased client independence and improved care experiences contributed to greater work satisfaction among professionals.

Second, inspired by the NASSS framework [14] and the implementation model by Meiland et al [15], our analysis categorized barriers to sustained use according to their level of impact and complexity and distinguished among deal-breakers, obstacles, and minor hurdles. Applying the same classification framework to facilitators as we did to barriers, we distinguished among key drivers, enablers, and minor boosters to assess their relative impact on the success of implementation. This classification emerged from analyzing both the 63% (12/19) of cases in which robot use was sustained and the 37% (7/19) of cases in which it was discontinued after 2 months.

Third, we categorized both barriers and facilitators not only by their level of impact on sustained use of the robot but also across 4 key domains: client characteristics, health care professional capabilities, contextual conditions, and organizational support. This domain-based classification allowed us to systematically examine how different factors shaped implementation success or led to discontinuation.

### Facilitators

The analysis revealed essential enabling factors required across all 4 domains for successful adaptive implementation. For clients, cognitive and sensory capabilities to process robot interactions, combined with a need for structure and predictable routines, were key drivers. This aligns with previous findings regarding the importance of matching technological capabilities to client needs in disability care [4]. The data showed that clients who could maintain attention during robot interactions and were receptive to neutral, consistent communication were more likely to benefit from sustained robot use.

For health care professionals, willingness to learn and adapt to new technology, ability to program basic client interactions, and available time for robot setup and maintenance emerged as critical factors. Using the robot, particularly its back-end programming interface that was essential for customization, emerged as a significant challenge, requiring both time and adaptability from professionals. Adequate training, dedicated programming time, and ongoing organizational support were identified as critical factors influencing the sustained use of the technology. These findings align with those of recent studies [8,9,13] stressing the importance of health care professional engagement in successful implementation of social robots in long-term care settings, as well as findings of research highlighting the need to optimize interfaces for all end users, including staff [22]. Moreover, health care professionals consistently emphasized the importance of dedicated time for programming and ongoing adjustments to maintain effective robot use.

Contextual analysis demonstrated that clear integration with existing care routines was essential for sustained use. The data revealed that support from informal caregivers, often family members, enabled successful implementation. Sustained use was jeopardized by issues related to the technology. Moreover, successful implementation depended on appropriate timing relative to staff availability. This highlights how organizational instability and high staff turnover can directly undermine technology adoption as temporary staff lack the knowledge and commitment needed to maintain consistent robot use.

Organizational support through management commitment, dedicated implementation time, and training opportunities emerged as a key driver. Health care professionals specifically highlighted the importance of systematic team training and shared learning opportunities. Providing professionals with the capacity and time to engage with the technology fostered sustained use. Our findings suggest that such support shifts the perception of the robot from a burden to an opportunity to enhance care. This is especially critical in health care, where overstretched resources demand solutions that benefit not only clients but also caregivers [2]. Sustained use of social robots depends on their ability to deliver value to all actors involved, including clients and caregivers [23]. In particular for social robot Ivy, whose setup and functionality heavily rely on caregiver input, organizational support is necessary to ensure that it creates value for both groups.

### Barriers

The interviews revealed distinct categories of barriers affecting implementation success. Following the NASSS framework [14], barriers were classified as deal-breakers (complex), obstacles (complicated), or minor hurdles (simple). For clients, deal-breakers included complex and unpredictable care needs requiring frequent adjustments, along with severe cognitive limitations affecting comprehension. Obstacles included fluctuating health conditions affecting engagement, whereas minor hurdles involved technical difficulties with touch screen operation. These findings align with those of previous research highlighting the importance of matching technology capabilities to client characteristics [24]. In addition, our study underscores that, beyond matching technological features (button and text size), factors such as the pace of interaction—how much time a client has to respond—are key in ensuring successful and sustained use, particularly for people with intellectual disabilities with varying levels of cognitive and physical abilities.

For health care professionals, deal-breakers centered on insufficient time for programming and maintaining the robot combined with high staff turnover. Obstacles included inconsistent use across team members and challenges in programming complex interactions. Minor hurdles involved initial learning curves with the programming interface. These findings echo those of recent studies emphasizing the need for dedicated time and resources in health care technology implementation [25].

Contextual deal-breakers included unstable connectivity and poor implementation timing. Obstacles included coordination challenges during holiday periods, whereas minor hurdles involved occasional technical glitches. This underscores the importance of stable care environments for successful technology implementation.

At the organizational level, deal-breakers included lack of systematic support and divergence from implementation road maps. Obstacles included unclear implementation responsibilities and fragmented initiatives within organizations. In line with recent research, this highlights the critical role of organizational commitment and structured implementation approaches in achieving sustained use of social robots in care settings [12].

### Limitations and Strengths of This Study

Some limitations of this study should be considered. First, our analysis relied primarily on health care professional perspectives as client interviews across all 19 cases were not feasible due to cognitive limitations. Second, although our study investigated adaptive innovation during the execution and continuation phases of early adoption, the 2-month implementation period captured only initial adoption and early use, not long-term sustainability.

The strength of this study lies in its analysis of 19 cases across 6 different care organizations, encompassing both sustained use and discontinuation, which provided rich comparative insights. The inclusion of both successful and discontinued cases allowed for a nuanced understanding of facilitators and barriers across multiple care settings. Unlike much of the existing body of literature, which primarily consists of scoping or systematic reviews [25-28] and laboratory or controlled studies [29,30], this field study investigated adaptive robot implementation in 19 actual productive care environments over a duration of 2 months. This approach offered a unique opportunity to explore long-term real-world experiences and behavior, allowing us to go beyond an early pilot phase [7,10] and overcome issues of low external validity associated with hypothetical or controlled scenarios [29,30]. Field studies in this area and at this scale are scarce, especially those examining the execution and continuation phases of adaptive innovation implementation. By investigating 19 unique cases of robot deployment rather than focusing on a single robot, this research provides particularly valuable insights. Furthermore, the use of established theoretical frameworks (the NASSS framework and the implementation model by Meiland et al [15]) ensured a structured and complete analysis of the value of sustained use and related facilitators and barriers.

### Scientific, Clinical, and Societal Relevance of This Study

This study makes several important contributions to the field. First, it provides a novel analysis of sustained versus abandoned use of social robots within the adopter system domain, focusing on value for the 2 key end user groups: people with intellectual disabilities and professional caregivers in real-world care settings. The findings present user value of the technology, including improved daily structure, greater independence, and enhanced emotional well-being for people with intellectual disabilities and support for health care professionals in delivering efficient, person-centered care. Second, it presents classifications of facilitators and barriers across 4 critical domains: client characteristics, health care professional capabilities, contextual conditions, and organizational support. These findings advance both research and practice by offering concrete implementation guidance while illuminating crucial prerequisites for successful robot deployment in disability care at both the micro level focusing on end users and the meso level addressing organizational factors.

By examining the execution and continuation phases of implementation [15] rather than early-stage preconditions and preparation, this study provides unique insights based on actual user experiences over a 2-month period. The findings reveal how social robots can create distinct value—enhancing mental and social health for people with intellectual disabilities and complex needs while simultaneously reducing workload and improving care delivery efficiency for health care professionals.

This dual focus on value creation and implementation factors is particularly significant given projected global health care worker shortages [1]. While previous research on IATs has primarily emphasized client benefits [3], our findings demonstrate that successful implementation requires creating value for both end user groups. IATs can effectively address workforce challenges and meet the growing demands for disability care services only when they meaningfully support both clients and caregivers and when implementation factors for both groups are adequately considered. By studying implementation in real-world care environments with their inherent staffing pressures and organizational dynamics, our research offers high external validity regarding the dual value creation needed for both clients and caregivers. The classification of facilitators and barriers across 4 domains provides organizations with concrete guidance for identifying and addressing critical implementation factors for both end user groups.

These findings have broader societal implications for national care strategies addressing health care workforce shortages, suggesting that social robots could be integrated into national workforce planning as complementary tools that optimize staff capacity while maintaining care quality. Indeed, health care internationally is experiencing rapid growth in social robot adoption, with evidence showing their utility in patient-facing roles—supporting tasks such as patient triage, telemedicine, and companionship—as well as in home settings for rehabilitation, medication management, and physical assistance [31]. However, our evidence of mixed implementation outcomes—with discontinuation occurring due to complex client needs, insufficient staff programming time, and unstable organizational contexts—indicates that policy frameworks should position social robotics as targeted solutions within mixed-care models, with funding structures that account for the training and implementation support necessary for success.

### Implications for Practice

Our findings provide several practical implications for long-term care organizations implementing social robots for people with intellectual disabilities. First, careful client selection is crucial. Organizations should focus on individuals with sufficient cognitive capabilities and predictable care needs to ensure meaningful and effective interactions with social robots. Second, health care professionals must be provided with dedicated time and resources to manage robot programming and maintenance. Without these, the integration of such technology into care routines may face significant challenges. Third, the timing of implementation must take into account both staff availability and client well-being. Properly aligning these factors can help avoid nonadoption or disruptions in robot use. Findings show that social robot Ivy can contribute to well-being, whereas a certain threshold of client well-being is required to accept the robot in the first place. For clients with complex or unstable care needs, organizations should develop flexible programming protocols that allow for quick adjustment of robot interactions during challenging periods—such as temporarily simplifying interactions rather than discontinuing use entirely. The programming interface could incorporate *crisis mode* templates that maintain basic support functions while reducing cognitive demands. In addition, training staff to recognize early indicators of client distress and make corresponding robot adjustments helps maintain continuity of care while respecting fluctuating client capacities. Fourth, organizations should deliver systematic training and support to entire care teams. This ensures that all staff members are equipped with the knowledge and skills needed to prevent disruptions of maintenance and frequency of use, thereby maximizing the value of social robot use. Finally, regular evaluation and adjustment of robot interactions are essential for sustained use. Continuous monitoring and fine-tuning help maintain engagement and ensure that the robots continue to meet the evolving needs of both clients and staff. These practical implications can guide organizations in creating optimal conditions for successful robot implementation in long-term care settings.

### Recommendations for Future Studies

Continued research is needed in 3 key areas. First, as studies have shown a high risk of dropout when using technology in care settings [32], longitudinal studies beyond the initial 2-month implementation period should evaluate sustained robot use and integration into care practices. Second, mixed methods studies directly investigating people with intellectual disabilities’ perspectives by combining qualitative data (such as observations or caregiver-assisted input during interviews) with quantitative interaction data would provide more comprehensive insights into robot usability and impact. Future work incorporating direct client feedback could provide a more complete evaluation. Essential metrics should include frequency of use, types of interactions, and objective measures of client engagement. Third, participatory design studies involving clients, care professionals, and technologists are needed to systematically improve both the robot’s client interface and the professional programming platform. In addition, studies investigating organizational implementation strategies can show how to optimize social robot integration at scale. In this study, we interviewed health care professionals that are directly involved in client care, resulting in a strong focus on microlevel factors related to clients, employees, and the specific context of implementation. Nonetheless, our findings also highlight organizational insights, suggesting that dedicated implementation teams and streamlined training programs may enhance the sustained use of service robots across diverse care environments. Future meso-level research should examine managerial strategies and organizational activities that enable health care professionals to seamlessly integrate social robots into existing care routines.

### Conclusions

Building on growing evidence supporting the potential of social robots in disability care [4,6-8], this study demonstrates that sustained use of social robot Ivy can create value for both people with intellectual disabilities and health care professionals when implemented under appropriate conditions. Success requires careful attention to user needs and preferences, client characteristics, health care professional capabilities, contextual factors, and organizational support. The classification of facilitators and barriers provides structured guidance for health care organizations implementing social robots for people with intellectual disabilities.

## Abbreviations

IAT: intelligent assistive technology
NASSS: nonadoption, abandonment, scale-up, spread, and sustainability

## Data Availability

The datasets generated or analyzed during this study are not publicly available due privacy restrictions established by the participating healthcare organizations, but are available from the corresponding author on reasonable request. Although requests for data access may be directed to the corresponding author, approval would require consent from all participating healthcare organizations.
